# From the Raw Materials to the Bottled Product: Influence of the Entire Production Process on the Organoleptic Profile of Industrial Beers

**DOI:** 10.3390/foods11203215

**Published:** 2022-10-14

**Authors:** Ana Belén Díaz, Enrique Durán-Guerrero, Cristina Lasanta, Remedios Castro

**Affiliations:** 1 Chemical Engineering and Food Technology Department, Faculty of Sciences-IVAGRO, University of Cadiz, Agrifood Campus of International Excellence (CeiA3), Polígono Río San Pedro, s/n, 11510 Puerto Real, Cadiz, Spain; 2 Analytical Chemistry Department, Faculty of Sciences-IVAGRO, University of Cadiz, Agrifood Campus of International Excellence (CeiA3), Polígono Río San Pedro, s/n, 11510 Puerto Real, Cadiz, Spain

**Keywords:** brewing, maturation, aging, spoilage microorganisms, sensory properties

## Abstract

In the past few years, there has been a growing demand by consumers for more complex beers with distinctive organoleptic profiles. The yeast, raw material (barley or other cereals), hops, and water used add to the major processing stages involved in the brewing process, including malting, mashing, boiling, fermentation, and aging, to significantly determine the sensory profile of the final product. Recent literature on this subject has paid special attention to the impact attributable to the processing conditions and to the fermentation yeast strains used on the aromatic compounds that are found in consumer-ready beers. However, no review papers are available on the specific influence of each of the factors that may affect beer organoleptic characteristics. This review, therefore, focuses on the effect that raw material, as well as the rest of the processes other than alcoholic fermentation, have on the organoleptic profile of beers. Such effect may alter beer aromatic compounds, foaming head, taste, or mouthfeel, among other things. Moreover, the presence of spoilage microorganisms that might lead to consumers’ rejection because of their impact on the beers’ sensory properties has also been investigated.

## 1. Introduction

Beer is one of the most popular and commonly consumed alcoholic beverages worldwide. However, a remarkable transition has been taking place regarding consumersʹ preference for traditional ‘tasteless’ beers, to more complex craft beers, with a growing quota of consumers being interested in new beer styles that exhibit novel sensory characteristics [1]. In addition, a growing consumer segment, comprising people between 21 and 30 years old, seems to be interested in new beer tastes and is willing to pay for these tasty beers, even if more expensive [2]. For this reason, brewers and researchers are investigating the use of alternative raw materials and processing conditions over the different stages of beer brewing so that its organoleptic profile is enhanced [3].

**Figure 1:**
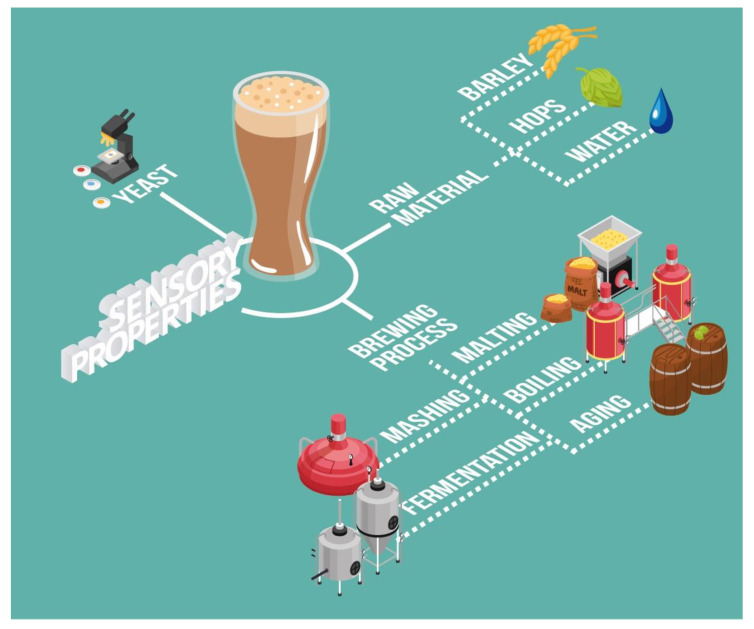
Main factors in the brewing process that have an influence on the sensory properties of beer.

Conventional brewing consists of four main processes: malting, during which enzyme production is activated and endosperm is modified; mashing, during which enzymes hydrolyze starch into fermentable sugars and proteins into aminoacids; boiling, during which resins undergo thermal isomerization and yield bitter taste; fermentation, during which sugars are converted into ethanol [4], and, finally, maturation and bottling (Figure 1). Beer has been traditionally made from malted barley (*Hordeum vulgare*), hops (*Humulus lupulus* L.), water, and yeast. It can also be supplemented with other cereals or sources of sugars known as adjuncts [5,6].

**Figure 2:**
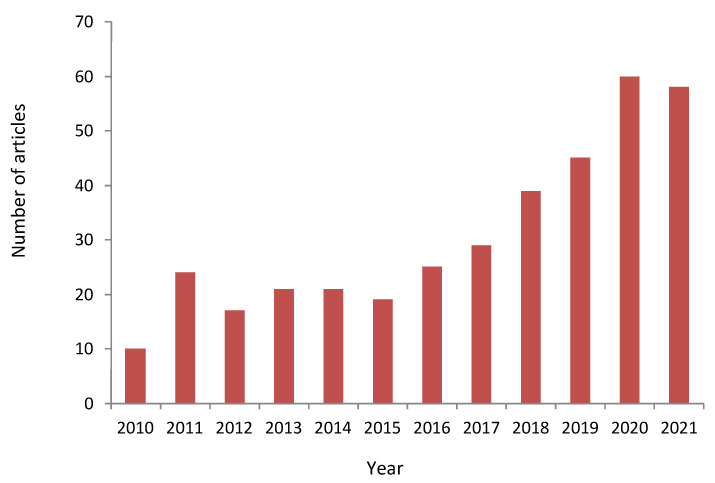
Evolution of the number of works published in Scopus using “beer” and “sensory analysis” as keywords.

The sensory characteristics of beers play an important role in consumers’ acceptance or rejection. As a consequence of this, the number of articles published regarding beer, as well as the number of sensory studies, have increased significantly in the last few years (Figure 2). Beer properties are affected by the variety of barley, yeast, and hops used. Hops provide beers with fruity, spicy, resinous, floral, and wood aromas [7,8].

Regardless of the Bavarian Purity Law and other country-specific legislations, grain blends and new hop varieties, as well as fruit and vegetables, have been incorporated in recent years into beer brewing in order to modify its sensory profile [9,10]. Such sensory profile is also influenced by the different processing stages involved in the whole brewing practice, including beer maturation and aging. In addition to this, bottle re-fermentation increases beer’s effervescence and, given its low oxygen levels due to yeast consumption, the volatile compounds that are associated with off-flavors may be developed [11,12].

It should be noted that between 1000 to 2000 compounds have been found in beers, including alcohols, esters, ketones, aldehydes, organic acids, carboxylic acids, sulfur compounds, phenols, amines, etc. [13,14]. These components are the result of complex reactions that involve a large variety of compounds. Among these, volatile compounds seem to have a key role regarding the aromatic profile of beer, while non-volatile ones, including anthocyanins and phenolic components, affect other sensory attributes such as color, foaming, taste, or mouthfeel, which are also considered as relevant contributors to the quality of beer [15]. It is well known that the abundance of a large number of aroma-active compounds is associated with specific fermentation conditions and to the particular yeast strain being used, which may yield interesting aroma components, such as higher alcohols or esters [16,17]. Other nitrogenous compounds, which may include proteins, polypeptides, amino acids, etc., are also key factors, because they contribute to beer flavor and drinkability, as well as to haze formation, foam stability, and color [18,19]. From a nutritional point of view, beer is rich in carbohydrates, amino acids, vitamins, minerals, and phenolic compounds [20]. The main polyphenols present in beer are flavonoids, tannins, proanthocyanidins, and amino phenolic compounds. These come from the malt and hops used for the brewing and significantly affect the color, flavor, and stability of the final beer [21].

Given beer’s considerable concentration of ethanol (0.5–10%), the presence of bitter compounds from hops (~17–55 ppm iso-α-acids), its low pH (3.8–4.7), and its high CO_2_ concentration (~0.5% *w/w*), it represents an inhospitable environment for most microorganisms [22]. However, some spoilage microorganisms, including Gram-positive and Gram-negative bacteria, as well as wild yeasts, are able to grow and cause some undesirable changes in beer’s sensory profile. *Lactobacillus* and *Pediococcus* are considered to be the most hazardous bacteria for beer production processes, as they account for around 70% of the microbial spoilage incidents [23].

All these facts considered, it seems rather clear that, in order to produce beer with a variety of sensory profiles that meet current consumers’ preferences, brewers may opt for a diversity of raw materials as well as adjuncts, adjust their brewing procedures, or select different yeast strains. The alcoholic fermentation process, being perhaps the most influential factor regarding the sensory characteristics of the final beer, has been investigated in a previous review [24]. Thus, the present review intends to delve into the rest of the potential factors that may affect the organoleptic profile of beers. Such factors have been identified as the main raw materials (barley, water, and hops), as well as the rest of the processes, other than the alcoholic fermentation, involved in beer production—specifically malting, mashing, boiling, maturation, and aging. Any likely effects from a variety of spoilage microorganisms have also been taken into consideration

## 2. Barley, Malt and Malting

Barley (*Hordeum vulgare* L.) is the most commonly used cereal in beer production, and its endosperm represents the main tissue of the grain, which is mainly composed of starch granules (62.7% of the total grain weight) embedded in a protein matrix [25,26]. Other cereals, such as wheat, rye, oat, triticale, sorghum, maize, etc., can also be used as raw material for beer production [27], as long as we are aware that this procedure may affect the sensory properties of the final beer. It is true that the usage of other cereals may confer beer with new properties and organoleptic features. However, at the same time, the incorporation of new cereals that do not contain the necessary enzymes may involve certain technological issues related to mash lautering, beer filtration, extract recovery, or production forecasting and scaling [28]. As an example, let us mention wheat beer, which is especially consumed in Bavaria and Austria and is characterized by clove-like, banana-like, vanilla, and fresh fruit scents [29,30]. Sorghum beers are described as one of the most subtle beers with regard to their sensory properties and mild taste [31]. Another study where different proportions of sorghum were used concluded that these beers have a lower acetaldehyde and ester content, and a greater proportion of higher alcohols [32]. The same authors described the beers that contained between 30 and 40% oat as better beverages in terms of aroma and taste purity than 100% malted barley beers, which contain lower amounts of lactones and higher alcohols, and a greater proportion of esters [33]. Other studies have concluded that different unmalted cereal adjuncts can replace malted barley at rates of up to 40–60% to produce beers with a sensory profile comparable to that of 100% malted barley beers [21,34].

Different barley genotypes lead to different chemical compositions, i.e., different enzymes and metabolites and, therefore, unequal results are expected from similar malting procedures [35,36]. Beers with higher fruity, floral, and grassy flavors are produced from Golden Promise barley—a classic British spring barley variety with a light malty flavor and beautiful mouthfeel—whereas other beers that are rich in toffee and toasted flavors, while exhibiting lower harshness or astringency, are obtained from Full Pint barley [37]. In this sense, CDC Copeland barley, a two-rowed malting barley, produces neutral flavors and pale colored beers. It has also been reported that, depending on the “terroir” where barley is grown, beers with different flavors can also be obtained [38].

The quality of the barley grain used as raw material is, therefore, another very important factor and it largely depends on agronomic practices, as well as on genetic and environmental variables [39]. Moreover, grain is required to present the adequate plumpness and kernel weight, with a high germination potential (≥95%) and the appropriate protein content. In this regard, barley’s protein content should be below 11%, otherwise the proteins that are soluble in wort may confer off-flavors to the final product. Moreover, high protein concentrations traditionally correlate with low carbohydrate levels and lower extract yields. The quality of barley may also be altered by microbial infections, being fungi the most commonly found microorganisms. These can infect barley while in the fields, especially during its wet growing season, while it may infect barley or malt while in storage under moist conditions. Primary gushing is associated with the use of defective malted barley, when harvested under wet conditions, while secondary gushing may be caused by solid particles, which may arise from various sources (dust polluted containers, faulty filtration, haze particles developing in aged beer, etc.), or adsorbed gas residues acting as nuclei for bubble formation. [40]. Primary gushing is derived from the use of malt made from barley spoiled by fungal contamination, especially by certain species of the genus *Fusarium*, which produces hydrophobins, a type of hydrophobic polypeptides that can induce this phenomenon through their interaction with CO_2_ molecules [41,42,43,44]. *Fusarium* spp. and other fungal pathogens of barley can produce mycotoxins that can survive the brewing process and can, therefore, be found in the final beer [16,44].

Malt contamination with *Aspergillus fumigatus* has been proven to be responsible for a noticeable rancid taste of beer [45]. Fungi growth on malt also has a negative effect on beer foam quality, because of the β-glucanases and xylanases produced that decrease the viscosity of the wort [16]. These and other negative aspects of the fungi that may grow on barley represent a hazard for the organoleptic properties of beer that may lead to consumers’ rejection [46]. Barley is also the vehicle for a variety of contaminating microorganisms other than fungi or molds and that may negatively affect the germination of barley prior to malting. *Clostridium* and *Bacillus* bacteria, which are generally associated with the production of butyric acid and sulfides, are amongst these other polluting organisms [47]. An excessive moisture level after completing the kilning or malting roasting process should be avoided if certain barley pollutants are to be prevented. In other words, proper storage and preservation procedures for the barley and the malted barley are crucial factors [16].

Barley is subjected to malting in order to solubilize the proteins and to break down the starch into fermentable sugars. Specific malting procedures also provide beer with characteristic colors and flavors [48]. During the malting process, the grains undergo chemical and structural changes that result in the generation of a number of precursors that will determine the organoleptic properties of the final beer, including its color, aroma, and flavor [49].

The malting process usually involves steeping, germination, and kilning. During the steeping process, cold water (10–15 °C) and oxygen are supplied into hygienic and calibrated kernels in order to maintain moisture levels at approximately 38–45% and promote the germination of the grains. At this stage, the grains’ endosperm cell walls and its proteins are broken through the action of certain enzymes, such as protease, amylase, or β-glucanase [50,51]. Different aspect of this process can be improved in several ways, as follows: using standardized seeds to achieve a uniform germination; using plump kernels to achieve maximum malt extract yields; and low protein content to attain higher extract levels and to enhance beer stability [52]. The quality of the malting process can be evaluated through ‘fine-grind’, which allows measurement of the soluble malt material, including fermentable sugars [53]. Other quality parameters used to evaluate the quality of the malting are kernel size fraction, kernel weight, protein contents, β-glucan, α-amylase activity, viscosity, and soluble nitrogen ratio [54]. Some malt-quality indicators are shown in Table 1 [55].

Germination is ended by drying the grains (moisture content down to 3–4%) through a gradual increment of the temperature from 50 to roughly 85 °C or more (kilning). The kilning process has a crucial impact on beer color and flavor [51], mainly as a result of Maillard reaction, which produces maltoxazine, maltol, isomaltol, and ethyl maltol, among other substances responsible for the caramel, bread, or cotton candy-like flavors in beer [56]. Therefore, through the control of the temperature, Maillard reaction can be adjusted to determine color formation and obtain different types of malt (base, caramel, special, amber, chocolate, or black) [26], which will result in variations of the compounds responsible for wort flavor and for the different organoleptic profiles of the final beers [36,57]. It should also be noted that the melanoidins generated through Maillard reactions may promote the growth of certain undesirable microorganisms. In fact, melanoidins have been used as antimicrobial agents against different pathogenic bacteria strains [58]. Apart from melanoidins, certain malt alkaloids, mainly hordatines, have also been proven to have an influence on beer flavor by increasing its astringency [59,60].

With the aim of reducing the carbon footprint associated with malt production, and given the large energy demand of kilning processes, some recent studies have focused on the usage of undried germinated (green) malt. Even though further studies would be required, the beers that have already been obtained through this methodology presented acceptable specifications with regard to color, pH, alcohol content, and foam stability [61].

Nowadays, brewers can use base malts (e.g., pilsen and pale), specialty malts, and roasted malts in order to produce beers with different flavors [37]. Uniquely flavored and colored beers have been obtained from specialty malts such as Crystal malt (also known as caramel malt), Brown malt, Cara malt, or Black malt, among others [62]. During the roasting operations when using these singular malts, some compounds, such as maltol and isomaltol, can be generated [63], which results in a sweet aroma of the final beer. These malts do not only add color, flavor, and antioxidant activity to wort and beer, but also affect the course of wort fermentation and the production of flavor-active yeast metabolites, such as vicinal diketones or esters [64]. Dark malts may improve foam stability and the mouthfeel of beer, presumably because of the presence of melanoidins [62].

Another aspect to be taken into consideration in relation to malt is that some lactic bacteria can be added in certain cases during the malting and mashing processes, because they compete against natural microflora, thus restricting the growth of certain harmful microorganisms such as fungi or bacteria [65]. Their preservation qualities are associated with the production of some organic acids, such as lactic or acetic acids, together with hydrogen peroxide and bacteriocins [66]. Moreover, bio-acidification increases malt yield and improves malt quality by decreasing its viscosity and shortening the time required for lautering and wort filtration. It has also been demonstrated to improve beer sensory quality and stability [67]. Lactic acid bacteria also produces other organoleptically active compounds besides lactic acid, including organic acids and esters, such as ethyl acetate, aldehydes, higher alcohols, ketones, phenolic, or heterocyclic compounds, and more [68,69,70].

## 3. Mashing and Wort

Mashing is an enzymatic process that produces sugars from malt to obtain wort, which is in turn fermented to produce beer. During this stage, the amylases, β-glucanases, and proteases degrade carbohydrates, β-glucans, and proteins, respectively. Their activities are affected by the temperature, pH, and composition of the solution, as well as by the processing time [71]. The action of these enzymes results in a final beer that contains a small amount of residual fermentable sugars (maltose being the most abundant one), a variable amount of dextrins, such as maltodextrin, and a small amount of peptides, which have an influence on the sensory properties and the palate fullness of the final beer.

Water is one of the most important ingredients during this mashing stage, because it represents most of the beer’s composition. The chemical composition of water, as well as the presence of pathogenic and/or non-pathogenic microorganisms, also has a considerable influence on the final result, so that it may even spoil beer to the point of rendering it unsuitable for human consumption [72].

The mineral composition of wort and beer, where the principal cations, such as calcium, magnesium, sodium, and potassium, as well as anions, such as sulfate, nitrate, phosphate, chlorides, and silicate, may also determine beer quality. The minor ions are iron, copper, zinc, and manganese [73]. Ions are necessary for the correct course of the fermentation process and for the growth of beneficial microorganisms [74], but also contribute directly to the flavor of beers as non-volatile taste-active compounds [75]. The mineral composition of the wort also depends on the nature of the raw materials [76]. Therefore, this factor must be taken into consideration when using cereals other tan malt.

As an example, Briggs et al. [26] established that the presence of calcium ions in the water used to make beer had a relevant influence on the mashing process and affected final beer flavor. According to Montanari et al. [73], calcium has the capacity to extract fine bittering principles from the hops and to reduce wort color, while sodium contributes to the perceived flavor of the beer by enhancing its sweetness. Other authors [77] have observed that “hard water” (with a high concentrations of salts; pH 8.47 ± 0.08) seemed to be a better extractor of the total carbohydrate content and B vitamins (riboflavin and niacin) than soft water (with a low concentrations of dissolved salts; pH 7.68 ± 0.23), whereas organic acid and iso-α-acid concentrations were not influenced by water pH values. It is a fact that the composition of wort has a great influence on the molecules that result from the fermentation process and, consequently, on the organoleptic profile of final beers. Therefore, as an example, wort sugar content levels and free amino nitrogen and lipids, as well as aeration [78] or temperature [79], are parameters that condition the subsequent production of aromatic esters by the microbiota [80,81]. Sucrose, fructose, glucose, maltose, maltotriose, and some dextrins, with maltose and maltotriose as the most abundant ones, are the main sugars that can be found in wort. Their concentrations depend on the characteristics of the barley and on the malting process [20].

Wort is also moderately rich in amino acids, peptides, and proteins [72]. Some amino acids are required for the healthy growing of yeast. Such amino acids, together with certain small peptides, constitute what is known as Free Amino Nitrogen (FAN). Total FAN is important for the fermentation (via yeast nutrition) and the stability of flavor. A high FAN content may affect beer flavor stability because of the production of vicinal diketones (VDK) such as diacetyl and 2,3-pentanedione, through the differential utilization of amino acids (valine and isoleucine, respectively) by yeast, which may provide beer with a butter- or butterscotch-like flavor or toffee-like flavor, respectively [82].

The releasing of free amino nitrogen and reducing sugars during the mashing stage contributes to a minor set of flavor precursors that can develop during the Maillard reaction, principally during wort boiling [83]. They are transformed during the mashing stage and through the metabolism of the yeasts during the fermentation stage into other new substances that contribute to the organoleptic profile of beer [17,84].

During mashing, a key cascade reaction is also initiated, where the products from lipid oxidation generate hydroperoxides that form active volatile compounds [83].

On the other hand, during this stage, unmalted adjuncts such as rice, wheat, corn, honey, or fruit can be added as an alternative cost-efficient source of extract that enables the production of innovative products that increase the content of bioactive compounds and generate unique flavors and bitterness and improve mouthfeel [5,85,86]. It has been observed that when rice is used in the brewing process, it provides neutral, clean, and dry sensory characteristics, whereas adding corn results in a fuller mouthfeel [87].

More sour, grainy, and sweet corn aroma beers were obtained when 60% torrefied maize was added to the wort [34]. The addition of unmalted barley at up to 50% resulted in beers with a preference rating that was comparable, with regard to odor and taste, to that of all-malt beers [88]. In contrast, when the added unmalted barley reached 90%, it resulted in more astringent beers, while 100% unmalted barley produced final beers with less body and mouthfeel [89].

Certain extracts from medicinal plants can also be added to the wort in order to produce beers with unique sensory characteristics and an increased concentration of various bioactive compounds, such as phenols [90].

## 4. Hops

Resins and essential oils can be found in the lupulin glands of female hop flowers, which, even when used in small amounts, contribute to bitterness and aroma (sensorially characterized by descriptors such as ‘fruity’, ‘floral’, ‘spicy’, ‘herbal’, or ‘woody’) [91,92]. In fact, hops are the main ingredient responsible for the bitterness of beer because of their polyphenols and α-acids contributions [93,94,95]. Hops contain a complex mixture of volatile compounds (essential oils), among which linalool, geraniol, and 4-methyl-4-sulfanylpentan-2-one are of particular importance [96].

Hop varieties can be classified as aroma hops, dual-purpose hops (aromatic and bitter), and bittering hops (very bitter) [97]. Saaz and the rest of the “noble hops”—Hallertauer Mittelfrüh, Tettnang, and Spalt—belong to the first category and are traditionally used for pilsners and lagers produced in the Bavaria and Bohemia regions. Another Saaz aroma hop, Styrian Goldings, is often preferred for Belgian-style ales. Bitter (high α-acid) or dual-purpose hops such as Citra, Centennial, Cascade, or Amarillo, among others, are typically used for American IPAs [98].

During wort boiling, the humulones (α-acids) that are found in the soft resins of hops are isomerized into isohumulones, which are the main components responsible for the bitterness of beer [99]. It has also been recently observed that the oxidized forms of humulones, humulinones that are present in dry-hopped and hop-forward beers, can also contribute to beer bitterness [100].

During wort boiling, the majority of the volatiles derived from hops are lost through evaporation. Thus, by the late addition of multiple dosages, we can obtain beers with hop aroma but without any extra hop bitterness. So, for a less bitter beer, hops can be added toward the end of the wort boiling stage, or to the whirlpool (late hopping) or to green and bright beer (dry-hopping) [101]. The flavor descriptors that are most often detected in late hopped beers are spicy, noble, herbal, woody, and, to a lesser extent, estery or fruity. Dry-hopping consists of the cold extraction of volatile and non-volatile hop compounds. This technique is widely employed by brewers to increase the aroma and stability of beer flavor [102]. The descriptors that are most frequently found in dry-hopped beers are floral, citrus, or pine [103,104]. Unlike in boiling hopping, dry-hopping does not allow for the thermal isomerization of the α-acids into iso-α-acids, which makes beer more prone to microbial instability [105]. Recent investigations on the microbial contamination hazards associated with dry-hopping techniques have detected spore-forming bacteria such as *Bacillus* spp., as well as Enterobacteriaceae, yeast, and fungi [106].

There is also evidence that the amylolytic enzymes present in hops can biochemically modify dry-hopping beer, which may lead to the degradation of long-chain, unfermentable dextrins into fermentable sugars [107]. This increase in fermentable sugars can, in the presence of yeast, give rise to a slow secondary fermentation, which is referred to as “hop creep” [108]. “Hop creep” represents a problem for brewers, because it modifies the specific density, flavor profile, and alcohol content of beers. Bruner et al. [109] revealed that hop creep resulted in 1.06% (*v/v*) alcohol increments in dry-hopped lager beers and 0.88% (*v/v*) in ale ones, over 30 day periods.

Beer aroma can also be modified by adding pure aroma hop extract [110]. Moreover, the addition of hop extracts to unhoped beer has been demonstrated to improve mouthfeel and fullness while increasing the bitter perception of beer [111]. Hop extracts are also commonly added for extra bitterness and to obtain a greater content of aromatic compounds from the different stages of the brewing process [112].

Even though hops have been extensively used since ancient times, they are susceptible to being replaced by other substances that can also provide those molecules responsible for the bitterness of beer, such as artichoke, carqueja, etc. The resulting beers have a similar sensory acceptance to that of commercial beers [113]. While hop oils contribute to beer flavor, the biotransformation of its glycosides [114] not only adds new flavors to the final beer but also plays a significant role in beer flavor stability [115,116].

The type and relative proportions of the molecules provided by the hops (hop bitter acids, phenolic acids, polyphenolic compounds, or volatile compounds) will vary depending on the hop variety. So, once again, genetics plays a relevant role in the process and determines the resulting sensory characteristics, especially with regard to the bitterness and aroma of the final beer [94,95,110,117]. Together with its genetics, the maturity level of hops will also determine the kind of contribution that they make toward a particular flavor or aroma [118,119]. There are also non-volatile compounds in hops, including carboxylic acids, resins, amino acids, carbohydrates, and polyphenols, which are known to have an influence on the taste and mouthfeel characteristics of beer [101,114]. Regarding the polyphenolic fraction, the most important groups of low molecular weight polyphenols present in hops are usually hydroxybenzoic acids, hydroxycinnamic acids, proanthocyanidins, monomeric flavanols, free flavanols, quercetin, kaempferol, and xanthohumol. The composition of polyphenols in beers are, once again, significantly determined by the hop variety used for brewing [120]. Furthermore, their concentration as well as the iso-α-acids content in the wort also varies with the temperature at which the hops are boiled, the boiling time, and the time of hopping [93,121,122,123]. These iso-α-acids exhibit antimicrobial activity, which means that they can inhibit the growth of some of the contaminating microorganisms that spoil the flavor of beer, acting therefore as preservatives [124]. In fact, the most common microbes responsible for beer spoilage are Gram-positive bacteria, which can actually be inhibited by hops. However, hops do not have the capacity to inhibit the growth of Gram-negative bacteria, such as *Pectinatus frisingensis*, *Pectinatus cerevisiiphilus*, or *Megasphera cerevisiae* [125,126]. Table 2 includes the most frequent microorganisms responsible for beer spoilage.

## 5. Maturation, Storage, and Bottling

Beer is an unstable product whose composition can change during storage and bottling [36] through different types of reaction. Table 3 shows the effects of maturation storage and bottling on the sensory properties of beer.

During the maturation phase, some off-flavor compounds from previous stages may reduce their concentrations and facilitate the production of a more balanced product. The bitterness provided by the hops and by some polyphenols such as gallic acid, flavonoids, and tannins, is also dependent on the specific conditions under which this phase takes place. Generally, during maturation, bitterness decreases and sweetness increases. Nevertheless, the extent to which this phenomenon occurs depends on a number of factors, including the type of beer [93]. In the case of lager beers, certain aromatic changes may take place during storage, together with a linear decrease in bitterness, because of the degradation of isohumulones and/or humulinones, and an increment of sweet aroma, toffee flavor, cardboard taint, and ribes off-flavor [143,144].

Certain compounds such as the furfural extracted from wood, and the esters generated by the esterification reactions that take place between alcohols—mainly ethanol and acids—during beer aging in wood change their concentrations, which increases beer bitterness as greater amounts of tannins are extracted from the wood [93]. Another aspect that should be considered during this particular maturation is that different microorganisms can contribute with different compounds to beer, but their presence will depend on the state and type of wood used for the aging [145]. For example, lambic beer matures in wooden casks, and yeasts such as *Brettanomyces bruxellensis*, *Brettanomyces anomalous*, and *Pichia membranifaciens*; acetic acid bacteria; and the LAB *Pediococcus damnosus* and *Lactobacillus brevis*, among others, play an important role in the process because they contribute to the typical Brett flavor of lambic beer, characterized by spicy and medicinal notes, and also fruity and floral ones. Thus, the ester-synthesizing activity of Brettanomyces contributes to the production of various ethyl esters, such as ethyl caproate or ethyl caprylate, that contribute to floral notes, at concentrations significantly higher than those found in other beers. In addition, the Brettanomyces yeast species that contain a superoxide dismutase enzyme with vinyl phenol reductase activity can form 4-ethylphenol and 4-ethylguaiacol, which are responsible for spicy and medicinal notes. Brettanomyces can also produce isovaleric acid from leucine, and this acid is responsible for sweaty and cheesy flavors, and may also produce mousy off-flavors that are associated with 2-ethyltetrahydropyridine and 2-acetyltetrahydropyridine. The presence of acetic acid and lactic acid bacteria also contributes to the high concentrations of ethyl acetate and ethyl lactate. In addition, acetoin, which is produced by AAB species through the utilization of lactate, may contribute to undesirable buttery notes [146,147].

It is also known that, after bottling, beer flavor is affected by certain chemical reactions that lead to instability, being an indicator of the increment of sensory-active aldehydes, which are generated in the sequence of radical reactions initiated by reactive oxygen species [149,150]. These aldehydes are also produced during mashing and wort boiling, but they decrease during the fermentation stage. It has been demonstrated that hop polyphenols slow down the sensory deterioration of pale lager beer as they suppress the formation of sensory-active aldehydes.

A traditional method to achieve beer carbonation consists of bottle re-fermentation, which is initiated by adding yeast and fermentable carbohydrates. As a result of yeast multiplication, carbonation increases and the concentration of flavor-active compounds is also affected, so that beer aroma and taste are also modified [11]. New flavors are produced as a result of the yeast activity, which incorporates higher alcohols, esters, aldehydes, vicinal diketones, and sulfur compounds that have an influence on beer aroma [17]. So, there are certain yeast strains that produce phenolic flavors resembling clove, smoked meat, or medicinal odors, among others [153]. Furthermore, the increment of carbon dioxide concentrations enhances beer effervescence. An additional effect of bottling is the prevention of oxidative damage, as yeast consumes oxygen.

Another factor to take into account is the presence of contaminants from previous stages that may reach the beer storage phase. During this phase, these contaminants may develop and grow. They can come from the raw materials, such as hops or barley, the latter being considered the main source of potential contamination [125]. In general, these undesirable microorganisms that may be present in the finished beer are not considered pathogenic and, therefore, they do not represent a potential hazard for consumers. However, if they are not eliminated they may still spoil an entire batch of beer [46,145]. The most common way that these contaminating microorganisms alter beer taste is by producing metabolites and other associated by-products, such as methyl mercaptan, dimethyl sulfoxide, or hydrogen sulfide, among others (which will differ depending on the species involved), at concentration levels that would allow a negative impact on the desired characteristics of the target beer. Certain traits in bottled beer, such as misshaped cans due to over-carbonation, turbidity, visible yeast colonies, superficial films, excessive viscosity, or some off-flavors, may act as indicators of a possible contamination [145]. Thus, some bacteria from the *Lactobacilli* genus provide lactic flavor, while others, such as *Pectinatus frisingensis*, *Pectinatus cerevisiiphilus*, or *Megasphaera cerevisiae* can add acetic, manure, rotten egg, or cooked vegetable aromas to beer and make them totally unpalatable for consumers [45,126,145]. The genus *Lactobacilli* is considered to be the most common and best-known bacterial contaminant of beer. Even though only a limited number of species are able to survive the entire brewing process, this type of contamination is more common than one would expect [126]. *Pediococcus* is another important genus of contaminating bacteria, and they produce diacetyl and provide beer with a buttery flavor [126]. *Staphylococcus xylosus* is another contaminating bacterium detected in homebrewed beers (much less common in industrial beers) that makes beer taste like bitter almonds [148]. Not only bacteria but also certain yeasts can be considered as contaminants of beer. These include *Brettanomyces*, *Candida, Debaromyces*, *Pichia*, *Hanseniaspora*, *Kluyveromyces*, *Pichia*, and *Torulaspora* [47]. An additional problem derived from contamination is an excessive increment of CO_2_ that over pressurizes the packaging and may cause can bursting or bottle caps popping off [151].

There are some microorganisms, such as *Lactobacillus* bacteria (naturally found in barley), that can eliminate certain undesirable microorganisms thanks to the production of antimicrobial compounds that have the capacity to inhibit the growth of other bacteria or fungi [125]. On the other hand, certain compounds from hops (mainly iso-α-acids) can act as preservatives against Gram-positive bacteria [154], but not against *Lactobacillus brevis*—a bacterium whose presence causes turbidity and super-attenuation problems. Other components in beers, such as carbon dioxide or polyphenols, can also act as preservatives and minimize the problems associated with potential contaminations [155].

Beers may also undergo oxidation reactions that can alter their flavor. Higher alcohols, unsaturated fatty acids, amino acids, or proteins generated through Maillard reactions, as well as isohumulones, are some of the compounds involved in such reactions. In this regard, De Francesco et al. [156] found that the addition of some polyphenols-rich extracts to beer before its bottling resulted in more stable beers, while the usage of condensed green tea tannins proved to be ideal for prolonging beer shelf-life.

Finally, and with regard to the bottling stage, it should be mentioned that some high-quality beer brands still use corked bottles. In those cases, the presence of certain compounds derived from the microbiological spoilage of the cork, such as chloroanisoles, bromoanisoles, or chlorophenols, can cause an undesirable “musty” off-odor, even when found at really low concentration levels (ng/L) [157]. Apart from these compounds, geosmin, 2-methylisoborneol, 2-isopropyl-methoxypyrazine, or 2-isobutyl-methoxypyrazine may also be responsible for this type of unpleasant odor. The latter ones may come either from the water or from any of the other raw materials, although they may also come from the brewery itself [152].

As mentioned above, beer contains a large number of natural components that enhance its resistance against undesirable microbiological processes; nevertheless, good brewing practices, from the raw material to the bottled beer, should be followed if high quality products are to be produced. Certain bottling practices, such as purging the bottle with CO_2_ prior to bottling, using antioxidants such as sulfur dioxide or ascorbic acid, adding arginine to inhibit the Maillard reaction, preventing oxygen from entering the bottles by means of efficient stoppers, limiting light exposure through the use of brown bottles, etc., are some of the measures to be considered in order to avoid any undesirable chemical and microbiological alteration of bottled beers.

## 6. Conclusions

The main raw materials for beer production (barley, water, and hops) have a significant impact on the sensory properties of beers. Different barley genotypes have been demonstrated to produce beers with different aroma profile, ranging from fruity and floral aromas to roasted ones. In this respect, some brewers have used specialty malts, which not only add color and flavor, but also have an influence on the course of the fermentation and production of flavor-active yeast metabolites. Protein content in barley is another factor to be monitored, as it can be responsible for the presence of off-flavors in the final product. During the kilning process, on the other hand, germinated barley is subjected to high temperatures that have a considerable impact on the final beer color and flavor as a consequence of the Maillard reactions. These reactions provide beer with certain compounds responsible for the emergence of caramel, bread, or cotton-like flavors. Water, as the major component of beer, may contain certain metal ions that also have a definite influence on beer flavor. The contribution to beer sensory profile from hops largely depends on the hop variety used and on its maturity level. Hops provide volatile compounds and alpha-acids to the wort, the latter ones being isomerized during the boiling stage into isohumulones, which are the main components responsible for beer bitter taste. In order to avoid hop bitterness while providing beer with hop aroma, hops can be added at the end of the boiling stage or after fermentation. This should allow for some of the most common aromatic descriptors, such as floral, citrus, or pine, to be present in the final beer sensorial profile. Alternatively, pure hop extract can be added to improve mouthfeel, fullness, and an increased perception of beer bitterness. Generally, during beer maturation, the aromatic profile of beer changes as bitterness decreases and sweetness increases. Other processes, including aging in wood barrels or bottle refermentation for beer carbonation, have also been demonstrated to change the aroma and flavor of beers. Special attention must be paid to the spoilage microorganisms that may develop during the whole brewing process, because they may definitely affect the sensory properties of beers.

## Figures and Tables

**Table 1:** Malt-quality indicators.

Quality Indicator	Recommended Values
Protein content	<10.8%
Kolbach index	38–34%
Extract content	>82%
Extract difference	1.2–1.8%
Viscosity	<1.55 mPa·s
β-glucan in wort	<300 mg/L
Wort color	<3.4 EBC

**Table 2:** Most common spoilage microorganisms in beer.

	Microorganisms		Compounds Produced	Spoilage Effect	References
Mold	*Aspergillus* sp.	*A. fumigatus*	Mycotoxins	Rancid taste, roughness, stale and moldy flavor	[125,127]
	*Fusarium* sp.	*F. graminearum* *F. culmorum*	Mycotoxins, hydrophobins, and hydrophobic polypeptides	Gushing	[41,43,46,125]
Yeast	*Brettanomyces* sp.	*B. bruxellensis*	Acetic acid, highly volatile phenolic compounds	Sweat, smoke, and cheese flavors	[128]
		*B. anomalus*	Tetrahydropyridines	Mousy off-flavor	[129,130]
	*Megasphaera* sp.	*M. cerevisiae*	Butyric acid, acetic, caproic, isovaleric and valeric acids, acetoin and hydrogen sulphide	Turbidity and off flavors (hydrogen sulphide)	[125]
	*Saccharomyces* sp.	*S. cerevisiae var. diastaticus*	Extracellular glucoamylase	Phenolic off-flavors, overcarbonation, and weakened body	[131,132]
	*Wickerhamomyces* sp.	Wickerhamomyces anomalus	Phenyl ethanol, ethyl propanoate, 2-phenylethyl acetate, and ethyl acetate	Solvent-like aroma	[133]
Bacteria Gram +	*Clostridium* sp.	*C. acetobutylicum*, *C. butyricum*, *C. pasteurianum*, *C.thermosaccharolyticum*	Butyric, propionic, valeric, caproic acids, sulfur compounds	Cheesy, buttery, putrid, and rancid aroma	[16,47,134,135]
	*Lactobacillus* sp.	*L. brevis*, *L. acetotolerans*, *L. casei*, *L. plantarum* *L. lindneri*	Lactic acid, acetic acid, and diacetyl	Buttery’ taste and oily mouthfeel, Turbidity and super-attenuation problems	[136,137,138,139,140]
	*Pediococcus* sp.	*P. damnosus*	Lactic acid, diacetyl	Sediments, reduced foam stability, sarcina sickness	[125]
Bacteria Gram −	*Acetobacter* sp.	*A. aceti*, *A. hansenii*, *A. liquefaciens*, *A. pasteurianus*	Acetic acid	Ropiness, sour and vinegary flavor	[126,141]
	*Gluconobacter* sp.	*G. oxydans*	Acetic acid	Cidery note, sour and vinegary flavor	[142]
	*Pectinatus* sp.	*P. frisingensis*, *P. cerevisiiphilus*	Acetic acid, propionic acid, acetoin, hydrogen sulfide, and methyl mercaptan	Rotten egg, cooked vegetable aromas	[141]

**Table 3:** Maturation, storage, and bottling effects that affect the sensory properties of beer.

Stage	Effect	References
Maturation and storage	Extracting wood compounds derived from maturation in oak casks Reducing some off-flavor compounds from previous stages Generally, reducing bitterness and increasing sweetness Increasing volatile compounds Producing microbial compounds that alter beer taste, such as methyl mercaptan, dimethyl sulfoxide, hydrogen sulfide, etc., that promote carbonation, turbidity, superficial films, and excessive viscosity Generating compounds derived from oxidation, including higher alcohols, unsaturated fatty acids, amino acids, and proteins that modify beer flavor	[45,93,126,143,144,145,146,147,148]
Bottling	Generating sensory-active aldehydes Producing “musty” off-odor derived from cork microbial spoilage or water and other raw materials Increasing the CO_2_ derived from the development of contaminants	[47,149,150,151,152]
Bottle re-fermentation	Increasing carbonation Promoting effervescence Generating new flavors Reducing oxidation products	[11,17,153]

## Data Availability

Not applicable.
